# Anti-PD-1/PD-L1 Antibody Therapy for Pretreated Advanced or Metastatic Nonsmall Cell Lung Carcinomas and the Correlation between PD-L1 Expression and Treatment Effectiveness: An Update Meta-Analysis of Randomized Clinical Trials

**DOI:** 10.1155/2018/3820956

**Published:** 2018-09-24

**Authors:** Qiuling Zhao, Ruixiang Xie, Shen Lin, Xiang You, Xiuhua Weng

**Affiliations:** ^1^Department of Pharmacy, Fujian Provincial Cancer Hospital, Fuzhou 350000, Fujian Province, China; ^2^College of Pharmacy, Fujian Medical University, Fuzhou 350000, Fujian Province, China; ^3^Department of Pharmacy, First Affiliated Hospital of Fujian Medical University, Fuzhou 350000, Fujian Province, China

## Abstract

**Purpose:**

This meta-analysis systematically evaluated the efficacy and safety of anti-PD-1/PD-L1 antibodies for pretreated advanced or metastatic nonsmall cell lung cancer (NSCLC) and investigated the correlation between PD-L1 expression levels and effectiveness of anti-PD-1/PD-L1 antibody.

**Methods:**

The methodology was based on the Preferred Reporting Items for Systematic Reviews and Meta Analyses (PRISMA) and the Cochrane Collaboration guidelines.

**Results:**

Our research included five randomized-controlled trials involving 3,025 patients. We compared anti-PD-1/PD-L1 antibodies (nivolumab, pembrolizumab, and atezolizumab) with docetaxel in pretreated patients with advanced or metastatic NSCLC. The pooled hazard ratio (HR) for overall survival (OS) and progression-free survival (PFS) was 0.69 (95%CI: 0.63-0.75, P<0.0001, and Ph=0.67) and 0.87 (95%CI: 0.81-0.94, P=0.0004, and Ph=0.11), respectively. Meanwhile, the pooled risk ratio (RR) for objective response rate (ORR) was 1.53 (95% CI: 1.16-2.01, P=0.003, and Ph=0.03) in all patients. Subgroup analyses showed that anti-PD-1/PD-L1 treatment significantly improved OS in patients with PD-L1 expression at any level, even in patients with PD-L1<1%. The RR for occurrence of grades 3 to 5 treatment-related adverse effects was 0.23 (95% CI: 0.15–0.36, and P<0.001).

**Conclusion:**

OS, PFS, and ORR were significantly more improved for patients treated with anti-PD-1/PD-L1 antibodies than for those treated with docetaxel. Anti-PD-1/PD-L1 therapy resulted in longer OS than docetaxel, regardless of PD-L1 expression; however, higher PD-L1 levels were likely to correlate with better outcome. Anti-PD-1/PD-L1 antibodies also had a better safety profile than docetaxel.

## 1. Introduction

Lung cancer represents the main cause of cancer-related mortality worldwide, [1, 2] with nonsmall cell lung cancer (NSCLC) accounting for 85% of lung cancers. Over 60% of newly diagnosed patients exhibit either locally advanced or metastatic disease, both with poor prognosis and with high mortality [3].

Patients with previously treated, advanced, or metastatic NSCLC are difficult to treat, with systemic cytotoxic chemotherapy (e.g., docetaxel) having only modest benefits. In recent years, epidermal growth factor receptor (EGFR) inhibitor development and application has shown significant benefits for advanced or metastatic EGFR-positive NSCLC patients, [4–8] though progress is generally evident after about 9 to 13 months of treatment. [9]

Immunotherapy is a relatively new paradigm for the treatment of NSCLC. The programmed death-1 (PD-1) receptor, expressed by activated T-cells, is engaged by the tumor-expressed ligands PD-L1 and PD-L2 to reduce T-cell activation and facilitate tumor immune escape. [10–12] PD-1/PD-L1 inhibitors for treatment of various advanced or metastatic melanomas and NSCLC are currently at different phases of clinical development [13].

Several inhibitors (i.e., nivolumab, pembrolizumab, and atezolizumab) targeting the PD-1 immune checkpoint pathway have been developed and approved by the United States Food and Drug Administration (USFDA) for the treatment of NSCLC. Compared with docetaxel, Nivolumab, a fully humanized IgG4 PD-1 inhibitor, showed significantly better overall survival (OS) and response rates (RR) in advanced squamous NSCLC, regardless of PD-L1 expression level [14]. In another randomized open-label trial, nivolumab showed better efficacy than docetaxel, based on the PD-L1 expression level [15]. In a Phase 3 study, PD-1 positive pretreated NSCLC patients treated with Pembrolizumab, a high affinity humanized IgG4 monoclonal antibody targeting PD-1, had better OS than patients treated with docetaxel [16]. In a trial by Rittmeyer et al. [17], Atezolizumab, an engineered IgG anti-PD-L1 antibody, improved survival compared to docetaxel, regardless of PD-L1 expression. Most clinical trials results show favorable survival outcomes for advanced NSCLC patients treated with anti-PD-1/PD-L1 antibodies than for those treated with conventional chemotherapy. However, a systematic evaluation of the overall efficiency and safety of anti-PD-1/PD-L1 antibodies for advanced NSCLC patients proved insufficient, especially regarding patient selection.

In the 2017 updates (Version 4), the NCCN Panel recommended that PD-L1 levels did not instruct the guidelines for treatment with some PD-1/PD-L1 agents, while other PD-1/PD-L1 agents were approved restrictively for patients with PD-L1 expression level ≥1%. Thus, the question remains whether PD-L1 expression should serve as predictor and guide for patient selection.

The aim of this meta-analysis is to further evaluate the efficacy and safety of anti-PD-1/PD-L1 agents in advanced NSCLC patients. A subgroup analysis was performed to determine the correlation between PD-L1 expression level and clinical outcome and to establish guidelines for PD-L1 antibody treatment in patients with low or negative PD-L1 levels.

## 2. Methodology 

This meta-analysis was performed in conformity with the PRISMA (Preferred Reporting Items for Systematic Reviews and Meta Analyses) [18] and Cochrane Collaboration guidelines [19].

### 2.1. Search Strategy

We performed a literature search of PubMed, Embase, and Cochrane Library electronic databases, using a combination of the terms “Carcinoma, Nonsmall-Cell Lung” [MeSH] or “NSCLC” and “PD-1” or “PD-L1” and “nivolumab” or “pembrolizumab,” or “atezolizumab.” The last search was performed on March 20th, 2017. No restrictions for language or publication year were set in the search.

### 2.2. Selection Criteria

The criteria for study inclusion were as follows:

(1) Prospective randomized-controlled trials (RCTs) designed for PD-1/PD-L1 inhibitor therapy for patients with advanced or metastatic NSCLC that had been previously treated.

(2) Published efficacy and safety measures reported and correlated to PD-L1 expression levels.

### 2.3. Data Extraction

Two reviewers (Q.L.Z. and X.H.W.) independently reviewed all abstracts, obtained full-text reports, and extracted data into separate databases. Disagreements were resolved through team discussion. For each study, the following information was extracted: first author's name, year of publication, trial phase, number of randomized patients, treatment strategies, clinical outcomes, PD-L1 status, hazard ratio (HR) for overall survival (OS) and progression-free survival (PFS) and their 95% confidence intervals (CIs), objective response rate (ORR), PD-L1 expression level, and overall grades 3-5 adverse events (AEs) and per grades 3-5 AEs.

### 2.4. Outcome Measures

The primary endpoint was overall survival rate. Secondary endpoints included PFS, proportion of patients with an objective response rate (ORR), and safety. The analyzed safety outcomes were grade 3-5 adverse events (AEs), including fatigue, decreased appetite, nausea, vomiting, diarrhea, constipation, anemia, neutropenia, and febrile neutropenia.

### 2.5. Qualitative Assessment

The 5-item Jadad scale was used to assess the quality of clinical trials and the calculated score was based on randomization, double-blinding, and reported withdrawals (Table 1) [20].

### 2.6. Data Analysis

All outcomes were pooled using RevMan 5.3 (Nordic Cochrane centre). Our analyses pooled HR with 95% CIs for OS and PFS and risk ratios (RR) with 95% CIs for ORR and grade 3-5AEs. HR<1 favored the experimental group (anti-PD-L/PD-L1 antibodies) whereas HR>1 favored the control (docetaxel). For each objective response rate and grade 3-5 AEs, a risk ratio (RR) was calculated based on the absolute numbers of patients presenting the objective response and grade 3-5 AEs, respectively. RR for ORR and AEs<1 indicated a higher overall response rate and toxicity in the control (docetaxel).* P*<0.05 was considered statistically significant. We assessed heterogeneity using a ***χ***^2^ test with* P*<0.10 considered to be statistically significant. A fixed effect model was used when heterogeneity between studies was absent and a random effect model was used when heterogeneity was present. Subgroup analysis was calculated based on PD-L1 expression levels. Sensitivity analyses were used to estimate the effect of each individual study by removing one by one from analysis. Publication bias was assessed using funnel plots.

## 3. Results

### 3.1. Search Results and Population Characteristics

**Figure 1:**
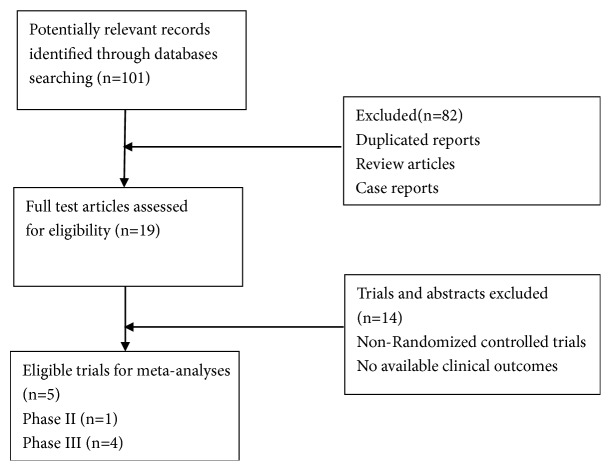
Studies eligible for inclusion in this meta-analysis.

A total of 101 relevant studies were electronically retrieved and 96 were excluded for the reasons shown in Figure 1.

Five published RCTs involving 3,025 patients with subgroup analysis assessing the efficacy and safety of PD-1/PD-L1 inhibitors in NSCLC were included in this meta-analysis [14–17, 21]. The baseline characteristics of each trial are listed in Table 2.

All included trials were considered high-quality data, as they were randomized when comparing anti-PD-1/PD-L1 agents (nivolumab, pembrolizumab, or atezolizumab) with docetaxel in the second or third line setting. Subgroup analyses, performed in all trials, explored the relationship between PD-L1 expression level and anti-PD-1/PD-L1 antibody efficacy.

### 3.2. Efficacy Outcomes

**Figure 2:**
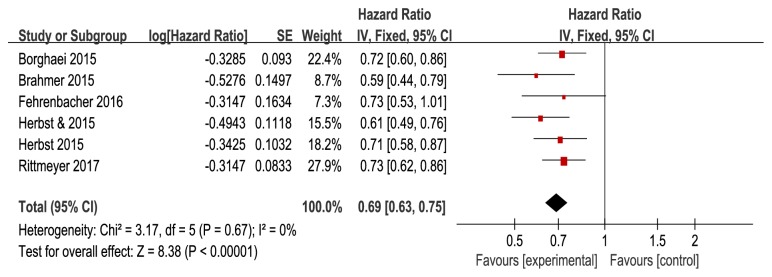
Forest plots of overall survival (OS).

**Figure 3:**
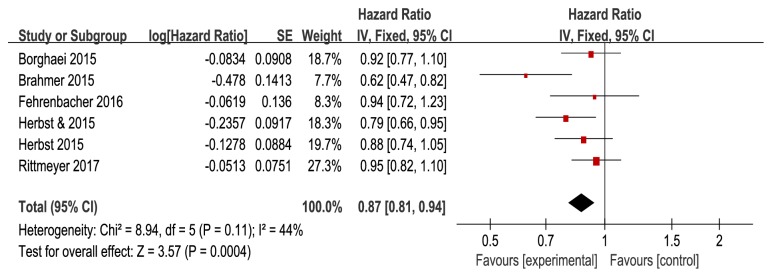
Forest plots of progression-free survival (PFS).

**Figure 4:**
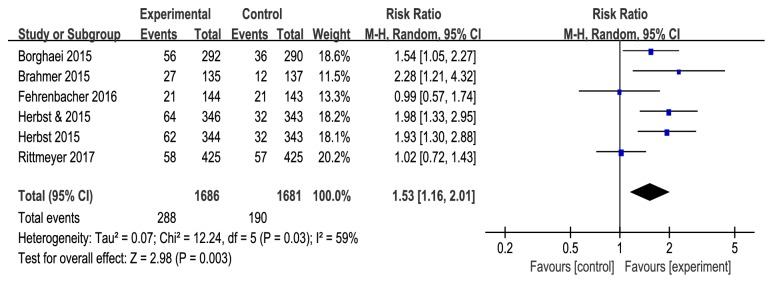
Forest plots of RR of objective response rate (ORR).

Pooled results showed that anti-PD-1/PD-L1 antibodies significantly improved the OS (HR=0.69, 95%CI: 0.63-0.75,* P*<0.0001, and P_h_=0.67) (Figure 2) and PFS (HR=0.87, 95%CI: 0.81-0.94,* P*=0.0004, and *P*_*h*_=0.11) (Figure 3) in all patients, when compared with docetaxel in a fixed effect model. Anti-PD-1/PD-L1 antibodies resulted in higher ORR than docetaxel (RR=1.53, 95% CI: 1.16-2.01,* P*=0.003, and* Ph*=0.03) (Figure 4). Moderate heterogeneity was observed between trials (*I*^*2*^=59%), and the pooled RR for ORR was determined using a random effect model.

**Figure 5:**
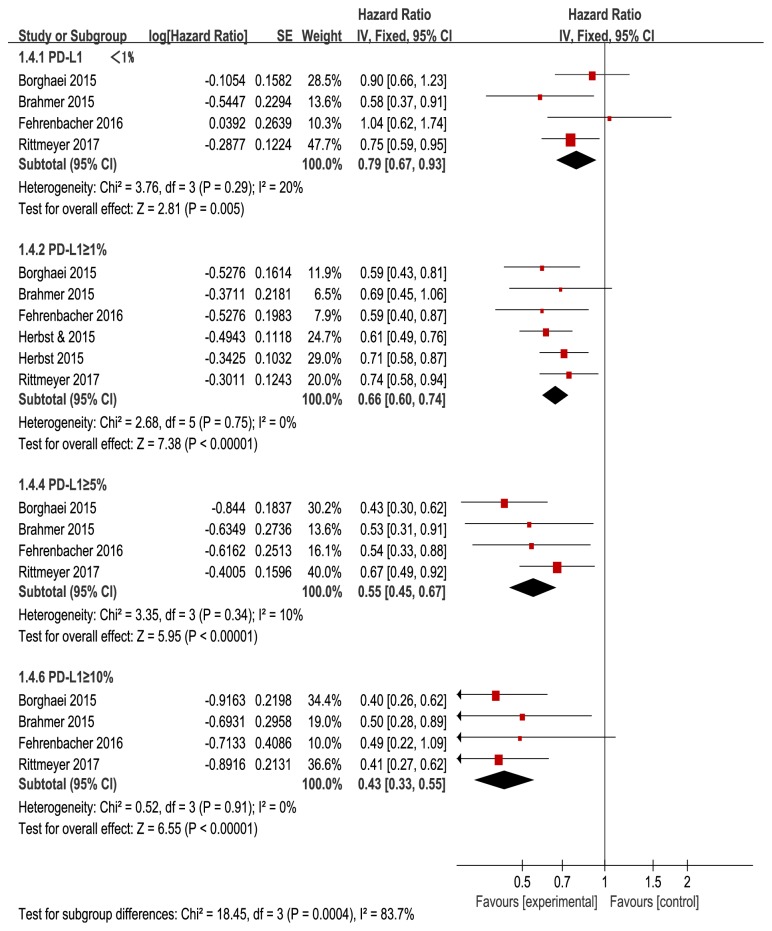
Forest plots of OS according to PD-L1 expression level.

Subgroup analyses showed that anti-PD-1/PD-L1 antibodies could result in longer OS (HR=0.79, 95% CI: 0.67–0.93,* P*=0.005, and *P*_*h*_=0.29) (Figure 5) than docetaxel in the population with PD-L1<1%. However, there was no difference in the PFS (HR=1.01, 95% CI: 0.86-1.17,* P*=0.95, and *P*_*h*_=0.16) and ORR (RR=0.82, 95% CI: 0.54-1.24,* P*=0.34, and *P*_*h*_=0.45)

However, in the PD-L1≥1% subgroup, PD-1/PD-L1 inhibitors significantly improved OS (HR=0.66, 95%CI: 0.60-0.74, and* P*<0.00001) (Figure 5), PFS (HR=0.83, 95%CI: 0.75-0.91;* P*<0.00001), and ORR (RR=1.87, 95%CI: 1.38-2.03;* P*<0.00001) when compared with docetaxel.

For the PD-L1≥5% population, the pooled HR for OS was 0.55 (95% CI: 0.45–0.67) (Figure 5) and for PFS, it was 0.66 (95% CI: 0.55–0.78). The RR of ORR was 2.12 (95% CI: 1.49–3.00). For the PD-L1≥10% subgroup, the pooled HR for OS was 0.43 (95% CI: 0.33–0.55) (Figure 5), for PFS, it was 0.57 (95% CI: 0.46–0.71), and the RR of ORR was 2.8 (95% CI: 1.82–4.29).

Therefore, this meta-analysis indicates that anti-PD-1/PD-L1 agents exhibited high efficacy in the treatment of advanced NSCLC. Anti-PD-1/PD-L1 therapy also had considerable activity for NSCLC and was superior to docetaxel in the PD-L1<1% population. PD-1/PD-L1 inhibitors tended to be associated with PD-L1 expression level. Higher PD-L1 expression was likely to be associated with increased benefit from anti-PD-1/PD-L1 agents.

### 3.3. Safety Outcomes

**Figure 6:**
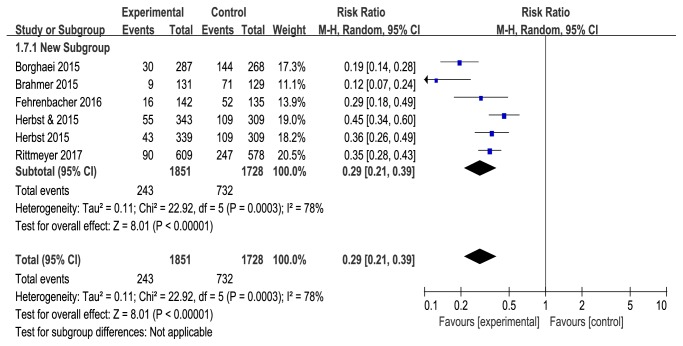
Forest plots of overall grades 3-5 adverse events (AEs).

The meta-analysis showed that the rates of overall grade 3-5 adverse events (AEs) for the anti-PD-1/PD-L1 therapy were significantly lower than those of docetaxel (Figure 6). For any grade 3-5 AEs, the rates of hematological AEs (anemia and neutropenia), febrile neutropenia, fatigue, and diarrhea were all significantly lower for anti-PD-1/PD-L1 antibodies than for docetaxel.

### 3.4. Sensitivity Analysis

The direction and magnitude of the statistical significance of the overall results were confirmed by this analysis. The benefit of anti-PD-1/PD-L1 antibodies on overall survival was maintained (HR 0.69–0.69) even when we changed the fixed-effect model to a random-effect model. Subsequently, an influence analysis was performed by excluding individual studies. The benefit of anti-PD-1/PD-L1 antibodies on OS (HR 0.71; 95% CI 0.63-0.79; P<0.001) did not vary regardless of study removal. The sensitivity analysis indicated the stability of all trials in the anti-PD-1/PD-L1 agents group.

### 3.5. Publication Bias

**Figure 7:**
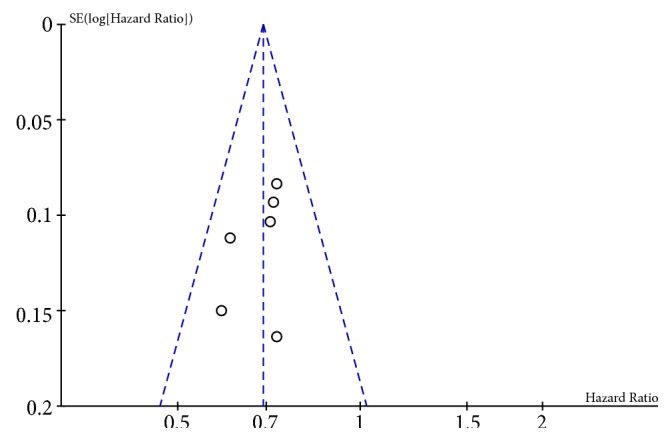
Funnel plot displays the publication bias for the five selected studies.

A funnel plot indicated no evidence of substantial publication bias (Figure 7).

## 4. Discussion

Blocking inhibitory immune checkpoints has recently gained interest as an immunological therapy for different kinds of cancer, especially advanced NSCLC. Binding of PD-1 to its ligands (PD-L1 and PD-L2), which are present on tumor cells, suppresses T-cell activation and results in immune response evasion [10, 22–24]. Therefore, blocking the PD-1 pathway by disrupting ligand-receptor binding is a promising effective approach for recovering antitumor T-cell mediated immunity. Nivolumab and pembrolizumab are highly selective humanized IgG4 monoclonal antibodies against PD-1. Atezolizumab is a humanized engineered IgG1 monoclonal antibody targeting PD-L1. Therefore, antibodies against PD-1 and PD-L1 are promising antitumor therapies as they can potentially reactivate the patient's own immune system.

In this study, a systematic meta-analysis of randomized clinical trials demonstrated the high efficacy and safety of anti-PD-1/PD-L1 antibodies for previously treated patients with advanced or metastatic NSCLC. Pooled results confirmed that anti-PD-1/PD-L1 agents significantly improved OS, PFS, and ORR in advanced or metastatic patients, both in the intention-to-treat population and in subgroups with PD-L1 expression level at 1% or more, 5% or more, and 10% or more. A high PD-L1 expression was likely to be associated with increased benefits. Furthermore PD-1/PD-L1 inhibitors also improved OS in the population with PD-L1<1%, which contradicts the guidelines for pembrolizumab administration only in PD-L1 positive patients (PD-L1≥1%). This discrepancy, may be attributable to the fact that the pembrolizumab trials excluded patients with PD-L1<1% patients or that the PD-L1 test might not accurately determine tumor PD-L1 levels. Other possible reasons may include heterogeneity of expression and sampling error, or that test samples predate earlier lines of therapy. Therefore, patients with PD-L1 expression levels just below and just above 1% will likely exhibit similar responses. Our results provide useful information for clinicians to inform their patients about treatment options for advanced NSCLC in the PD-L1<1% population. However, further research is needed to confirm these findings.

Subgroup analyses showed a trend toward a greater efficacy as PD-L1 expression level increased. In other words, patients that expressed the highest levels of PD-L1 derived the greatest benefit from anti-PD-1/PD-L1 therapy (PD-L1 expression≥1%: HR=0.66; ≥5%: HR=0.55; ≥10%: HR=0.43). Importantly, patients with PD-L1<1% (HR=0.79) also experienced OS longer than those treated with docetaxel without any evidence of statistical heterogeneity; however, PFS and ORR showed no difference. These results may imply that the benefit from PD-1/PD-L1 inhibitor versus docetaxel in pretreated advanced NSCLC is not limited to the PD-L1>1% population. More importantly, our findings could indicate a dose-effect relationship between the levels of PD-L1 expression and the potential benefit from PD-1/PD-L1 inhibitors. However, our meta-analyses included only five RTCs, and to further confirm these hypotheses, larger sample size studies are necessary.

Treatment-related adverse effects are an important evaluation index for any antitumor therapies. Our meta-analysis showed that anti-PD-1/PD-L1 antibodies had lower risk of total grade 3-5 adverse events than docetaxel. Pooled RR for total grade 3-5 adverse events was 0.29 (95%CI: 0.21-0.39, P<0.00001) compared with docetaxel, with statistical heterogeneity (*I*^2^=78%, *P*_*h*_=0.0003). The reason for heterogeneity could be because different PD-1/PD-L1 inhibitors (nivolumab, pembrolizumab, and atezolizumab) have potentially specific pharmaceutical characteristics. Treatment-related AEs of grade 3-5 were similar with those observed after docetaxel treatment and included decreased appetite, nausea, vomiting, and constipation. However, hematological AEs (anemia and neutropenia), febrile neutropenia, fatigue, and diarrhea were all significantly less common for anti-PD-1/PD-L1 agents. Only a small percentage of patients treated with anti-PD-1/PD-L1 agents reported immune-related adverse events, including hypothyroidism and pneumonitis, and with the use of appropriate protocol guidelines, these events were relieved. Our study showed that anti-PD-1/PD-L1 therapy is superior to docetaxel in clinic application and presents a lower risk for treatment-related adverse events.

In an era of personalized medicine using PD-1/PD-L1 inhibitors, predictors of response to therapy are important for making informed treatment decisions. PD-L1 expression may be an encouraging predictor for anti-PD-L1/PD-1 therapy in NSCLC, but standardizing PD-L1 testing has presented several problems. Different studies showed contradictory results regarding the relationship between drug efficacy and PD-L1 expression levels. Brahmer et al. [14] showed that PD-L1 expression was neither predictive nor prognostic of treatment efficacy in patients with squamous-cell lung cancer, whereas Borghaei et al. [15] demonstrated a strong predictive association between PD-L1 expression and nivolumab in advanced non-squamous cell lung cancer. Given the difference in histological features, controversy regarding NSCLC treatment persisted. Other factors regarding detection of PD-L1 expression further heightened the confusion. First, PD-L1 expression is dynamic and its expression in tumor samples before, during, or after previous treatment or immunotherapy could have affected PD-L1 immunohistochemistry (IHC) results [25] Second, each study used a different anti-PD-L1 IHC detection assay developed by different companies [26, 27]. For example, the Nivolumab trial used the anti–PD-L1 IHC antibody clone 28-8 (Dako, Glostrup, Denmark) and tumor staining for PD-L1 was assessed using different thresholds (1%, 5%, and 10%) to define positive results [14, 15]. Alternatively, in the pembrolizumab trial, the detection test used a different anti–PD-L1 Dako clone (22C3), set only two “positive” thresholds of tumor staining (1% and 50%), and the published data supporting a threshold of 50% or greater, for first line use [28]. Also, the atezolizumab trial used the anti–PD-L1—SP142 clone [17, 21]. These various methods and interpretations for PD-L1 IHC assessment could have resulted in the differences in PD-L1 expression standard threshold. Third, the cut-off value range to determine tumor PD-L1 expression status was wide according to the different studies. In some studies, IHC staining of more than 1% was defined as PD-L1 positive, but 5% and 10% were also used as cut-off criteria in other studies. These factors can generate confusion for clinical treatment and cause discrepancies among studies. As such, they could have influenced the results of our meta-analysis, to a certain extent.

Our analysis has some limitations. Due to the recent introduction of PD-L1 inhibitor therapy for pretreated advanced NSCLC, there were only five randomized clinical trials that investigated the efficacy and safety of anti-PD-1/PD-L1 antibodies, which limited the number of studies available for our meta-analyses. More randomized controlled trials with a larger sample size are needed to establish and replicate these clinical outcomes. Second, all five trials were liable to probable bias due to an open-label study design. Third, statistical heterogeneity was found when we pooled the objective response rate and the grade 3-5 adverse effect rates, respectively. Because of this, we used a random-effect model to pool outcomes. Since no evidence of substantial publication bias was found, these results indicated reliability to evaluate clinical outcomes.

In conclusion, we analyzed five RCTs and systemically verified favorable OS, PFS, and ORR of anti-PD-1/PD-L1 therapy for pretreated advanced or metastatic NSCLC and demonstrated higher efficacy and safety for these agents than for docetaxel. More importantly, the results of this meta-analysis suggested that anti-PD-1/PD-L1 antibodies could also improve overall survival even when PD-L1<1%, which has not been recommended by previous studies. Our results could be of great value in guiding selection of clinical therapeutic regimens. More prospective studies are necessary to confirm these results and to improve the optimal dosage for PD-1/PD-L1 inhibitors in NSCLC.

## Figures and Tables

**Table 1:** Jadad quality score of included studies.

Study	Randomization	Blinding	Reported withdrawals and dropouts	Overall score
Borghaei	2	0	1	3
Brahmer	2	0	1	3
Herbst	2	0	1	3
Fehrenbacher	2	0	1	3
Rittmeyer	2	0	1	3

**Table 2:** Baseline characteristics of RCTs included in the analysis.

Study	Year	Study type	Intervention	Treatment regimens	No. of patients
Brahmer	2015	Phase III	Nivolumab	3mg/kg ivgtt q2w	135
			Docetaxel	75mg/m^2^ ivgtt q3w	137
Borghaei	2015	Phase III	Nivolumab	3mg/kg ivgtt q2w	292
			Docetaxel	75mg/m^2^ ivgtt q3w	290
			Pembrolizumab	2mg/kg ivgtt q3w	344
Herbst	2015	Phase III	Pembrolizumab	10mg/kg ivgtt q3w	346
			Docetaxel	75mg/m^2^ ivgtt q3w	343
Fehrenbacher	2016	Phase II	Atezolizumab	1200mg ivgtt q3w	144
			Docetaxel	75mg/m^2^ ivgtt q3w	143
Rittmeyer	2017	Phase III	Atezolizumab	1200mg ivgtt q3w	425
			Docetaxel	75mg/m^2^ ivgtt q3w	425

## Data Availability

The data used to support the findings of this study are included within the article.
